# Impact of Extraction Conditions on the Phenolic Composition and Antioxidant Capacity of Grape Stem Extracts

**DOI:** 10.3390/antiox8120597

**Published:** 2019-11-28

**Authors:** Nerea Jiménez-Moreno, Francesca Volpe, Jose Antonio Moler, Irene Esparza, Carmen Ancín-Azpilicueta

**Affiliations:** 1 Department of Sciences, Universidad Pública de Navarra, Campus Arrosadía s/n, 31006 Pamplona, Spain; nerea.jimenez@unavarra.es (N.J.-M.); francesca.volpesainz@gmail.com (F.V.); 2 Department of Statistics and Operational Research, Universidad Pública de Navarra, Campus Arrosadía s/n, 31006 Pamplona, Spain; jmoler@unavarra.es; 3 Institute for Advanced Materials (InaMat), Universidad Pública de Navarra, 31006 Pamplona, Spain

**Keywords:** grape stem, mazuelo, bioactive compounds, phenolic extraction, green extraction

## Abstract

The use of grape stems for the extraction of bioactive compounds to be used in the pharmaceutical, food, and cosmetic industries is a promising objective. The aim of this work is to determine the influence of the different extraction conditions (temperature, ethanol concentration, and ratio of sample/solvent) on phenolic composition and antioxidant capacity of Mazuelo stem extracts. In general, the ethanol concentration of the extraction solvent was the factor that had the greatest influence on the extraction of different bioactive compounds. The greatest content of total phenolic compounds and the highest antioxidant activity of the extracts were obtained with 50% ethanol and at 40 °C. The most abundant compound found in the different extracts obtained from Mazuelo grape stem was (+)-catechin, but appreciable concentrations of gallic acid, a quercetin derivative, and stilbenes (trans-resveratrol and *trans*-ε-viniferin) were also extracted. Quercetin and malvidin-3-glucoside showed the highest correlation with the antioxidant capacity of the extracts, while stilbenes did not present such relation. The maximum concentration of gallic acid was extracted with water but the extraction of most of the compounds was maximum on using 50% ethanol. Consequently, the selection of the extraction method to be used will depend on the particular compound to be extracted in greatest quantity.

## 1. Introduction

According to figures from the International Organization of Vine and Wine (OIV), grape production in 2016 reached a total of 28,233,181 tons in Europe and, therefore, this sector has an important economic impact for many European countries. From this large grape production, approximately 75% is used for the winemaking [1]. As a result of this process, the wineries generate a huge amount of solid waste (skins, seeds, and stems) that cause a negative environmental impact and an important economic loss. Grape stems make up some 25% of the total waste generated, and it is the winemaking waste currently less characterized and used [2]. Grape stems are discarded during the destemming stage as their presence during vinification would introduce green tannins in the wine, as well as give undesirable herbaceous and vegetable aromas. Moreover, as they are rich in water and poor in sugars, they lower the alcoholic content of wine [3]. Traditionally, in some countries, grape stem has been used as fertilizer, for proteins extraction, and for the production of spirits [4]. The use of grape stems to produce fertilizers requires a previous extraction of polyphenols due to their phytotoxicity and antimicrobial activity, which could endanger the efficiency of the composting process [5]. However, the use of this waste for the extraction of bioactive compounds deserves further attention as it could be an important source of products with a high added value for different industries (food, cosmetics, pharmaceutical, etc.). Recently, some researchers have studied the composition of grape stems. However, there are still few research groups working in this field. Data currently available show that grape stems are rich in polyphenol compounds and, therefore, could become an important and cheap source of these bioactive compounds [6]. Specifically, grape stems are characterized by containing notable quantities of stilbenes such as *trans*-resveratrol and its derivatives. These phytochemicals are allowed as food ingredients and display important in vitro antioxidant effects [7,8]. The high antioxidant potential of grape stems is basically due to the presence of polyphenols, which have redox properties that allow them to act as reducing agents, singlet oxygen quenchers, hydrogen donors, metal chelators, and reductants of ferryl hemoglobin [9,10]. In fact, Apostolou et al. [11] showed that grape stem extracts are capable of inhibiting OH•^−^ and ROO•^−^ induced DNA damage and the growth of HEPG2 and HeLa cancer cells. Furthermore, Vázquez-Armenta et al. [12] found that the extracts of Red Globe and Mazuelo grape stems inhibited the adhesion of *Listeria monocytogenes* to food contact surfaces (polypropylene and stainless steel surfaces) by inhibiting the motility, and modifying its adhesive potential. Over the last few years, the antimicrobial potential in grape stems has been described with regard to pathogens such as *Staphylococcus aureus,* among others [13,14]. All of this makes it clear why there is interest and potential of these by-products for their use in the field of nutraceutical and functional food, among others.

However, to use the antioxidants extracted from grape stems in the field of food, nutraceuticals, or cosmetics, it is necessary to optimize the extraction processes by using non-toxic solvents and scalable methods. Most of the studies performed up to now used inappropriate solvents for food sector applications [15,16,17,18,19,20,21]. Some studies analyze the composition of extracts obtained with proper solvents for the food industry, but they did not study how the solvent influenced on the final composition of these extracts [22,23,24]. Other authors [25,26] studied the influence of the ethanol content and other factors, such as temperature and the time length of incubation, on the extraction of phenolic compounds and the antioxidant capacity of the extracts. However, in these studies they did not identify which phenolic compounds were extracted under the different experimental conditions, and this information is very interesting for selecting the optimal extraction method to use depending on the future application of the extract obtained.

For all these reasons, the aim of this work was to evaluate how the extraction conditions (temperature, ethanol concentration, and the ratio sample/solvent) influence both on the phenolic composition and on the antioxidant capacity of Mazuelo grape stem. This study also aims to contribute to the knowledge of grape stem composition, to understand how their most relevant components are extracted depending on the extraction conditions and to analyze the relationship between these components and the antioxidant capacity of the extracts. 

## 2. Materials and Methods

### 2.1. Samples

The samples used in this study were grape stems from Mazuelo variety collected from the 2016 harvest in Navarra, in the north of Spain. Grapes were manually removed and the resulting stems were dried in a stove (Ing Climas, Barcelona, Spain) at 25 °C until constant weight. Later, dried grape stems were milled in a coffee grinder (Moulinex, Ecully, France) and sieved in a sieve of 300 µm. The resulting powder had brown color and a homogeneous particle size.

### 2.2. Solvent Extraction

We investigated the influence of three process parameters (each one varying on different levels) on extraction of phenolic compounds from grape stems. These parameters were ethanol concentration (five levels, 0%, 25%, 50%, 75%, and 100%, *v*/*v*), solid/solvent ratio (two levels, 1:50 and 1:100, *w*/*v*) and extraction temperature (two levels, 25 °C and 40 °C). We carried out a full factorial design and all the 20 resulting combinations were performed in triplicate. To perform the extraction processes, adequate amounts of dry matter were weighed (0.5 g or 0.25 g depending on the solid/ solvent ratio used) and then, 25 mL of the corresponding extraction solvent were added. Each mixture was incubated for 24 h at 25 °C or 40 °C in a stove (Ing Climas, Barcelona, Spain) under orbital shaking (150 rpm). After the incubation, the extracts were centrifuged (8000 rpm for 15 min), filtered, and lyophilized. Once freeze-dried, the extracts were reconstituted with 3 mL of methanol, filtered through 0.45 μm PTFE syringe filters, and stored at −20 °C until further analyses.

### 2.3. Antioxidant Capacity Determination of the Extracts Obtained from Grape Stems

The antioxidant capacity of each extract was determined by three different methods: 2,2-diphenyl-1-pycrilhydracyl (DPPH) radical scavenging assay, 2,2′-azinobis (3-ethylbenzothiazoline-6-sulphonic acid) (ABTS) radical scavenging assay, and ferric ion reducing antioxidant power (FRAP). Each sample was analyzed in triplicate (three dilutions per sample) by each method, and results were expressed as mmol of Trolox per gram of dry matter (DM) of grape stem.

The DPPH assay used in this work is based on the method outlined by Brand-Williams et al. [27]. A standard solution of 24 mg of DPPH in 100 mL ethanol was prepared (DPPH radical). The DPPH stock solution was prepared by diluting the DPPH standard solution in methanol until obtaining an absorbance of 0.93 ± 0.04 at 515 nm. For the antioxidant capacity determination, 150 μL of grape stems extract (previously diluted 20 times with methanol in triplicate) were added to 2.85 mL of the DPPH stock solution and after 30 min in darkness, the absorbance at 515 nm was measured with a UV/Vis spectrometer (Jenway 7315, Staffordshire, UK). For the calibration curve, Trolox was used in different concentrations ranging from 0.05 mM to 0.80 mM.

The ABTS method used in this study is based on the method outlined by Re et al. [28]. Firstly, a solution of ABTS 7 mM with potassium persulfate 2.45 mM was prepared, and the mixture was left in darkness for 16 h (ABTS•^+^ radical cation). Calibration curve was made from a 5 mM solution of Trolox, ranging from 0.05 to 2.00 mM. The antioxidant capacity was determined by adding 30 μL of grape stem extract (previously diluted 20 times with methanol in triplicate) to 2.97 mL of ABTS•^+^ solution, and measuring the absorbance at 734 nm after 30 min in darkness. 

The FRAP method used is that proposed by Benzie and Strain [29]. This method is based on the reduction at low pH of the Fe^3+^-TPTZ complex to ferrous form in presence of antioxidants. Different concentrations of Trolox (0.05 mM–0.90 mM) were used for preparing the calibration curve. The absorbance of each sample was measured at 595 nm, after 30 min in darkness of 2.85 mL of FRAP mixture and 150 μL of extract (previously diluted 10 times with methanol in triplicate). 

### 2.4. Total Phenolic and Flavonoid Content Determination

Total phenolic content (TPC) was analyzed using the Folin–Ciocalteu method outlined by Singleton et al. [30]. The calibration curve was prepared with solutions of gallic acid at different concentrations ranging from 0.2 to 4 mM. Then, 0.1 mL of gallic acid standard or extract (previously diluted five times with methanol in triplicate), 0.5 mL of Folin–Ciocalteu reagent, 7.9 mL of deionized water and 1.5 mL of Na_2_CO_3_ were mixed, and the solution was left for 2 h in darkness. The absorbance was measured at 765 nm. Results were expressed as mmol of gallic acid per gram of dry matter of grape stem. 

The determination of flavonoids was based on the method described by Suman et al. [31]. To do so, 500 μL of extract were added to 500 μL of a solution of 2% AlCl_3_ in 5% acetic acid (*v*/*v*) and the absorbance at 420 nm was measured. For the calibration curve, quercetin was used as standard at different concentrations between 3–30 ppm. Results were expressed as mg of quercetin per gram of dry matter of grape stem. 

### 2.5. Identification and Quantification of Phenolic Compounds by HPLC-DAD

Analyses of the phenolic compounds were performed with a high-pressure liquid chromatograph (Waters, Milford, MA, USA) equipped with two 510 pumps, a 717 Plus autosampler, and a Photodiode Array 996 detector. A reverse phase column was used (Zorbax Eclipse Plus C18, 250 × 4.6 mm, particle size of 5 μm) at 30 °C. The instrument control and data processing were carried out with Empower 2.0 software (Waters, Milford, MA, USA). For the chromatographic analyses, a modified method of Barros et al. [16] was used. Two mobile phases, A (water: 85% formic acid, 99:1 *v*/*v*) and B (acetonitrile: 85% formic acid, 99:1 *v*/*v*) were used. The flow rate was 1 mL/min using the following linear gradient scheme (t in min; % A): (0; 95%), (15; 85%), (30; 30%), (22; 80%), (35, 70%), (45; 50%), (50, 5%), and (55, 95%). The injection volume was 20 μL. All the HPLC quality solvents were from Scharlab (Barcelona, Spain). The identification of the compounds was carried out by a double coincidence of the UV-Vis spectrum at the characteristic wavelength of each compound, and the retention time of its corresponding standard. For the identification of the phenolic compounds from the different extracts, methanol solutions were prepared of different standards. These standards were: Eight phenolic acids (syringic, vanillic, gallic, *p*-coumaric, caffeic, chlorogenic, and ferulic acids), five flavonols (quercetin, quercetin-3-glucoside, quercetin-3-glucuronide, rutin, and kaempferol), three flavanols ((+)-catechin, epicatechin, and epigallocatechin), two procyanidins (procyanidin B1 and B2), five flavones (luteolin, luteolin-7-rutinoside, apigenin, apigenin-7-glucoside, and apigenin-7-rutinoside), two flavanones (naringenin-7-rutinoside and naringenin-7-glucoside), three anthocyanins (cyanidin, cyanidin-3-rutinoside, and malvidin-3-glucoside), and three stilbenes (viniferin, resveratrol, and polydatin). All the standards used were from Sigma-Aldrich (Madrid, Spain), with the exception of malvidin-3-glucoside (Extrasynthese, Genay, France). From among all the standards prepared, eight phenolic compounds were detected in grape stem extracts. These compounds were gallic acid, catechin, quercetin, a quercetin derived compound (quantified as quercetin-3-glucoside), malvidin-3-glucoside, an unknown anthocyanin (quantified as malvidin-3-glucoside), resveratrol, and viniferin. Quantification was carried out using calibration curves for each compound analyzed. In all cases, the coefficient of linear correlation was *R*^2^ > 0.999.

**Figure 1:**
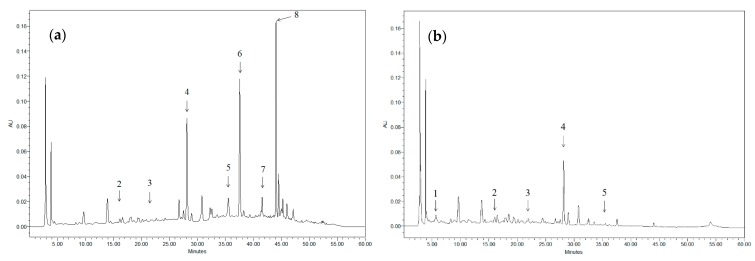
Chromatograms of grape stem extracts displayed at 324 nm: (**a**) obtained after incubation in 50% ethanol at 40 °C for 24 h (solid/solvent ratio 1:100); (**b**) obtained after incubation in water at 25 °C for 24 h (solid/solvent ratio 1:100). 1: Gallic acid; 2: Catechin; 3: Malvidin-3-glucoside; 4: Quercetin derivative; 5: Anthocyanin; 6: Resveratrol; 7: Quercetin; and 8: Viniferin.

Figure 1 shows, as an example, the chromatogram of two different extracts. Taking into account that the different compounds found in the extract have very distinct maximum absorption wavelengths (λ_max_), it is not possible to display all the existing peaks in the same chromatogram (for example, when anthocyanins are displayed, the rest of the compounds present in the sample cannot be seen). Nevertheless, although not all the compounds present in both samples are depicted at the selected wavelengths, it is possible to identify the most characteristic ones in each case. 

### 2.6. Statistical Analysis

Different statistical treatments were performed on the data. Firstly, the influence of the extraction variables (temperature, ratio, and ethanol content) on the antioxidant capacity and phenolic composition of the grape stem extracts was studied with a hierarchical linear model in order to incorporate repeated measurements in the study. Later, a principal components analysis (PCA) was applied with the aim of establishing relationships between the phenolic compounds present in the extracts and their antioxidant capacity. To perform this statistical treatment, all the observations of all the variables studied in the present study were used. Data analysis was performed with the packages implemented in the software R.3.2.0 (R core team, Free Software Foundation, Boston, MA, USA).

## 3. Results and Discussion

We investigated the influence of ethanol concentration, solid/solvent ratio, and temperature on the extraction of bioactive compounds from grape stems. We selected ethanol as extraction solvent, because it is generally recognized as safe (GRAS) for potential applications of the extracts in food or drug fields. The other two parameters to be studied, as well as the total duration of the extraction process (24 h), were selected based on published researches and on some preliminary trials conducted in our laboratory (data not shown).

Among the 20 extractions obtained, the extraction yields ranged between 14.6% (25 °C, ratio 1:50, 100% ethanol) to 37.6% (40 °C, 1:100, 75% ethanol). However, it is important to point out that these values are not directly related to the phenolic composition of the samples or to their antioxidant activity. This is because in the extracts, there could be different compounds with antioxidant capacity apart from polyphenols, as well as polyphenols or other compounds with a scant or null antioxidant activity.

### 3.1. Antioxidant Capacity, Total Phenolic and Total Flavonoid Content of the Different Extracts Obtained from Grape Stems

**Figure 2:**
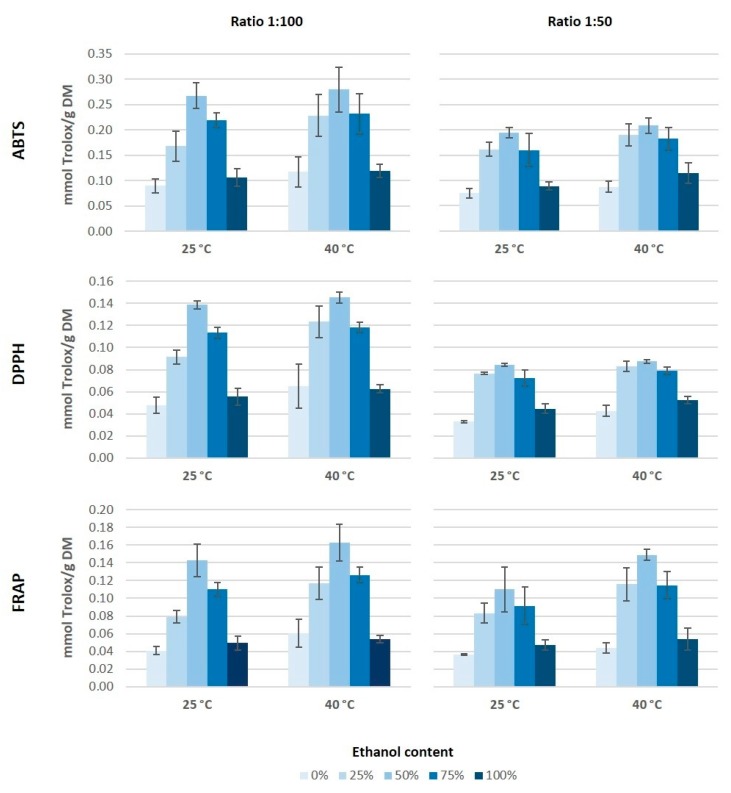
Antioxidant capacity of grape stem extract obtained measured by 2,2′-azinobis (3-ethylbenzothiazoline-6-sulphonic acid) (ABTS), 2,2-diphenyl-1-pycrilhydracyl (DPPH), and ferric ion reducing antioxidant power (FRAP) methods.

The antioxidant capacity and total phenolic content constitute a useful tool to determine the potential of an extract for its application in functional foods, cosmetics, nutraceutical, or any other field. The antioxidant capacity is the number of moles of free radical scavenged by an antioxidant testing solution that could lead to different results for the same radical. There is no universal method to measure the antioxidant capacity accurately because this estimation is strongly affected by the reactive oxygen species (ROS) or reactive nitrogen species (RNS) employed in the assay. For that reason, the antioxidant capacity of the different extracts was measured by three different assays: DPPH, ABTS, and FRAP (Figure 2). All of these methods are usually used to determine the antioxidant features of a sample, even though their action mechanisms are different. DPPH and ABTS measure the antiradical capacity of the sample, while the FRAP assay determines its reducing capacity [32].

According to the antioxidant capacity results, it would seem that both the ethanol concentration in the medium as well as the ratio solid/solvent have an influence on the extraction of antioxidant compounds from grape stems (Figure 2). The highest values of antioxidant capacity were obtained for the extracts obtained using 50% of ethanol and a 1:100 ratio, independently of the assay used. As for the temperature, it seems that an increase of temperature tends to generate higher values of antioxidant capacity. Moreover, it is noticeable that the highest values of antioxidant capacity were obtained by the ABTS method. The behavior pattern of antioxidant capacity obtained for the different extracts was similar through either ABTS, DPPH, or FRAP. However, the absolute value obtained by the ABTS assay was almost two-fold higher than the values obtained by the other two methods, which coincides with the results from Barros et al. [16]. On the other hand, the values of antioxidant capacity obtained in our extracts from Mazuelo grape stems were lower than those obtained by Barros et al. [16] from different Portuguese grape stems varieties. These authors found values of antioxidant capacity, between 30 and 70 mmol Trolox/100 g DM by ABTS assay and between 20 and 45 mmol Trolox/100 g DM when they used DPPH and FRAP methods. 

González-Centeno et al. [5] analyzed the antioxidant capacity of different Spanish varieties of grape stems and they obtained values between two and four times higher to those obtained in our study for Mazuelo stems. This large difference could be due to the grape variety and because these authors used a much more aggressive and less environmentally-friendly extraction method, as they carried out eight successive extractions with a mixture of acetone and water (80:20, *v*/*v*) followed by another three successive extractions with methanol and water (60:20, *v*/*v*). Conversely, other authors used combinations of ethanol and water as solvent extraction and they obtained values of antioxidant capacity slightly lower than those obtained in this study, probably because they used a lower ratio solid/solvent [25]. 

**Figure 3:**
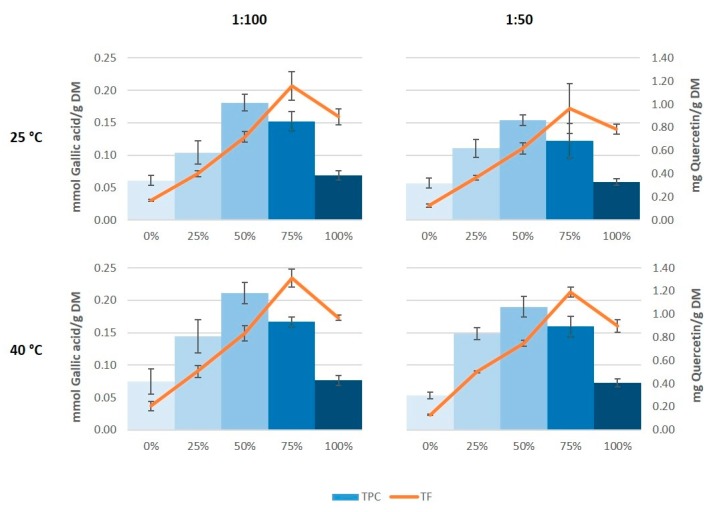
Total phenolic content (TPC) and total flavonoid content (TF) of the grape stem extracts.

The results of total phenolic content showed a very similar profile to those of antioxidant capacity (Figure 3). Thus, the maximum value of total phenolic content was found in the extracts obtained with 50% ethanol, and this was in the same order as the values obtained by Çetin et al. [33] in grape stem extracts obtained with 60% ethanol, and by González-Centeno et al. [5] in Merlot stem extracts treated with water, acetone, and methanol as extraction media. However, the maximum values of total flavonoids were obtained on using 75% ethanol as extraction solvent instead of 50% ethanol. Again, it also seems that on increasing the temperature somewhat higher values were generated both in the total phenolic content as well as in the total flavonoid content, which coincide with the results found by Domínguez-Perles et al. [25].

With the aim of determining how the different variables of extraction influenced statistically, and selecting the most determinant ones in order to maximize both the content of phenolic compounds as well as the antioxidant capacity of the extracts obtained, a hierarchical lineal model with fixed effects (temperature, ratio, and ethanol content) and random effects attributed to the replica was selected. The observed value of the replica is the average value of three measures and consequently, the effect of the repetition of measures was not considered in the model since it did not have significant variability. In Table 1 the estimations of the hierarchical model are shown. In this table only the significant interactions at 5% (*p* < 0.05) have been included.

The ethanol concentration was the only factor that had a significant influence on the TPC and on the antioxidant capacity of the extracts (Table 1). Other authors such as Domínguez-Perles et al. [25] and Karvela et al. [26] had also observed that the most determinant factor in the extraction of polyphenols from grape stems was the ethanol content in the extraction solvent. The extraction temperature only influenced on the antioxidant capacity when it was determined by DPPH assay. In addition, interactions between temperature and ethanol concentration were found in the values of total phenolic and flavonoids contents. These interactions could be explained by considering that at higher temperature, ethanol has higher capacity to solubilize phenolic compounds, and surface tension and solvent viscosity decreases with temperature, which will improve sample wetting and matrix penetration, respectively [34]. However, it is also likely that the two temperatures tested in this work were not different enough to produce significant differences between the treatments. It is advisable to be careful when increasing the extraction temperature, because some phenolic compounds could degrade easily. For example, *trans*-resveratrol is thermally decomposed at temperatures higher than 60 °C in 80% ethanol [35], and this stilbene is widely recognized as one of the most important compounds in grape stems. 

The ratio solid/solvent only had effect on the total flavonoid content (TF), being higher the extraction of these compounds when the 1:100 ratio was used (Table 1). In the same way, the ratio also influenced on the antioxidant capacity measured by means of the ABTS and DPPH assays, but only interacting with the ethanol concentration at 50%.

### 3.2. Identification and Quantification of Phenolic Compounds in the Different Extracts Obtained from Grape Stems

Table 2 compiles the chemical formulas, the retention time, and the maximum absorption wavelength of the different phenolic compounds detected in the grape stem extracts. 

Of all the compounds identified, (+)-catechin was the major compound in all the extracts, including the one which was obtained with water as extraction solvent (Table 3). Other authors also identified the (+)-catechin as the major compound in grape stem extracts coming from different *Vitis vinifera* varieties from Greece [11,15], Portugal [23], and Mexico [12]. The concentrations of catechin in the present study were similar to those found by the above-mentioned authors.

Catechin is present in the solid parts of grape bunches (grape stems, seeds, etc.) and is dissolved in wine during the maceration phase [36]. The highest extraction of catechin was reached when the percentage of ethanol was at 50%, and the increase in temperature favored a higher extraction of this compound. This compound has two centers of asymmetry in its structure and so it can give rise to four optically active forms and two racemic forms (catechin and epicatechin series). Among them, (+)-catechin and (−)-epicatechin are the most abundant in must and wine [37]. However, in this work (−)-epicatechin was not detected in Mazuelo stems. Some authors did identify this compound in grape stem from different varieties of red and white wines [21,23], while other authors only found it in some varieties and in much lower concentration to that of catechin [11,15]. 

The second most abundant component in the different extracts was a derivative of quercetin (Table 3). Among the possible derivatives of this compound, several authors identified quercetin-3-glucoside in their grape stem extracts [11,13,15,23], while others identified quercetin-3-glucuronide [38,39,40], or even both [41,42]. In the present study, this derivative has been quantified as quercetin-3-glucoside. However, taking into account that both quercetin-3-glucuronide as well as quercetin-3-glucoside showed very similar spectra and retention times in the chromatographic method used, it has not been possible to identify clearly which of them is present in the extracts obtained or if it is, in fact, a combination of both of them. For this reason, the value included in the table should not be considered as an absolute quantitative data, but simply as a useful value for comparing the different extracts. The quercetin derivative was extracted in greater amounts as the proportion of ethanol in the solvent extraction increased, reaching the maximum extraction when its percentage was between 50%–75% (Table 3). As for quercetin, its concentration in grape stem was much lower to that of its derivative, which coincides with the results found in the literature [11,15]. Unlike its derivative, quercetin was not extracted with water in any of the temperatures tested, and the maximum extraction of this compound was obtained with ethanol at 25%, decreasing as the percentage of ethanol increased (Table 3). This compound was hardly extracted when 100% ethanol was used. 

Gallic acid was the only compound that was extracted better by using water instead of hydroalcoholic mixtures as extraction solvent. The water extraction was similar in the two temperatures and ratios studied and the presence of ethanol lowered the extraction efficiency. In the work of Spatafora et al. [42], gallic acid was the only phenolic acid detected in Nero d’Avola and Frappato grape stems. In the present study, other phenolic acids such as syringic acid, vanillic acid, *p*-coumaric acid, caffeic acid, chlorogenic acid, or ferulic acid were explored without success. However, other authors have found some of them in different grape stem varieties, although in very low concentration [12,15,23].

As for the anthocyanins, malvidin-3-glucoside was identified in the extracts analyzed in the present study as well as another anthocyanin, which could not be identified as its retention time and its UV-Vis absorption spectrum did not coincide with any of the standards of anthocyanins analyzed (cyanidin, keracyanin). Very few studies identify anthocyanins in grape stems. Some authors such as Queiroz et al. [38] performed a semi-preparative analysis by HPLC of a grape stem extract and found malvidin-3-*O*-glucoside and malvidin-3-*O*-(6-*O*-caffeoyl)-glucoside. Likewise, Barros et al. [16], Días et al. [13] and Domínguez-Perlés et al. [40] identified both compounds along with malvidin-3-*O*-rutinoside in grape stem extracts of different varieties of red and white wines from Portugal. As can be seen on Table 3, the maximum extraction of both anthocyanins was reached with 50% de ethanol as extraction solvent. Silva et al. [23] also used a mixture of 50% of ethanol in their extractions but they did not detect anthocyanin in their samples. Consequently, despite the fact that the concentration of ethanol would seem to be the most influencing factor in the extraction of anthocyanin, it is important to consider other factors such as grape variety, incubation time, the ratio solid/liquid and temperature.

Finally, both *trans*-resveratrol as well as *trans*-ε-viniferin were detected in the different samples analyzed (Table 3). In the present study *trans*-resveratrol was not detected when water was used as extraction solvent, and with 25% of ethanol in the medium it was only extracted in the samples subjected to 40 °C. The extracts obtained with 50% of ethanol and at 40 °C showed the highest content of *trans*-resveratrol, being very similar to the samples obtained with 75% and 100% of ethanol. ε-viniferin, a dimer of resveratrol, showed an extraction tendency similar to that of resveratrol. Piñeiro et al. [43] also found a similar extraction behavior in both stilbenes against the ethanol percentage and temperature, in extracts from grape canes obtained in the pruning season. However, these authors found a total concentration of stilbenes higher than the values obtained in the present study, probably due to the use of ultrasounds that enhances the extracting effect of ethanol, as well as the different raw material used. Nevertheless, the results obtained in the present study were found within the range of concentrations described by Gouvinhas et al. [44] in red wine varieties (between 0.09 mg/g DM and 0.27 mg/g DM in the case of resveratrol, and between 0.12 mg/g DM and 5.82 mg/g DM in the case of ε-viniferin). Ewald et al. [45] analyzed the stilbene content of grapevine canes and grape cluster stems from different red and white grape varieties from Germany, and the predominant stilbenoids in either canes and stems were *trans*-ε-viniferin and *trans*-resveratrol. These are the most cited stilbenes in grape stem studies, although some authors such as Piñeiro et al. [46] also found piceatannol in grape stem extracts from 4 varieties of white wine grape and 11 varieties of red wine grape. Of the stilbenes found in grape stem, *trans*-resveratrol has been of great interest for researchers in recent years for its proven therapeutic anti-inflammatory and even its anti-cancerous effects [22,47,48]. 

Once again, a hierarchically linear model with randomness in the replicas was used in order to determine how the extraction variables influence on the individual phenolic compounds of the extracts. Table 4 shows the results obtained and, just as in the case of Table 2, only the significant interactions are shown (*p* < 0.05). The effect of ethanol on all the tested concentrations was clear, except at 100% ethanol, where catechin, quercetin-3-*O*-glucoside and malvidin-3-glucoside did not show differences with regard to 0% ethanol. In all cases temperature interacted with the ethanol, but especially when its concentration was at 50%. On the other hand, the ratio solid/solvent did not influence the extraction of the different compounds.

Consequently, considering these results along with those obtained by spectrophotometric methods, the extraction of polyphenols was maximum when ethanol was used at 50% and temperatures were at least, 40 °C, independently of the ratio solid/liquid used. The ratio solid/solvent hardly had any influence on the variables under study (it only had some on the total flavonoid content). Therefore, it seems reasonable to use the lowest of them (1:100), since adding more grape stem does not achieve either a higher concentration of phenolic compounds or a greater antioxidant capacity. Further studies would be necessary in order to evaluate if the temperature could be increased in order to attain better results, taking into account the possible degradation effects on the phenolic compounds and the added difficulty that would entail the process scaling. So, at this point it should be noted that these would be the optimal extraction conditions in order to maximize antioxidant capacity, total phenolic content and the concentration of flavonoids and stilbenes. However, in the cases where it is desirable to extract high quantities of gallic acid from the grape stem, the most adequate extraction medium would be water, also at 40 °C. 

### 3.3. Relationship between Phenolic Composition of the Grape Stem Extracts and Their Antioxidant Capacity

**Figure 4:**
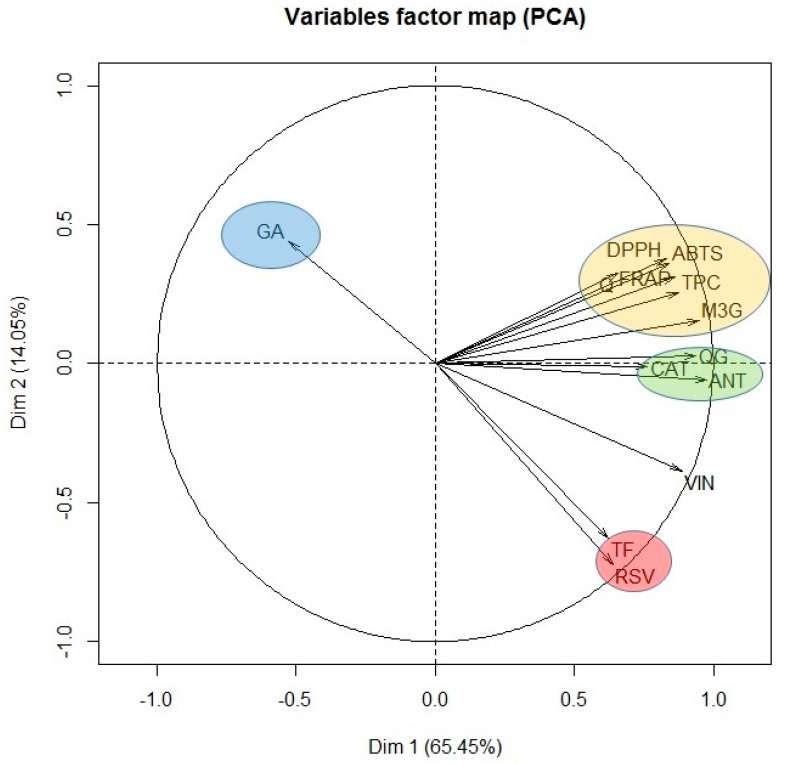
Principal component analysis of all the variables assayed: ABTS, DPPH, FRAP, TPC, total flavonoid content (TF), gallic acid (GA), catechin (CAT), quercetin (Q), quercetin-3-glucoside (QG), malvidin-3-glucoside (M3G), unknown anthocyanin (ANT), resveratrol (RSV), and viniferin (VIN).

Finally, in view of the results obtained, it seems that the antioxidant capacity of the extract obtained when only water was used as extraction solvent, was due especially to its content of gallic acid, catechin and the quercetin derivative. However, when 100% ethanol was used, although important concentrations of stilbenes were found, the antioxidant activity was low. Therefore, it seems that the stilbenes do not contribute very much to the antioxidant activity of the grape stem. In order to confirm this hypothesis and with the aim of identifying the most important components influencing the antioxidant capacity of grape stems, a PCA was carried out with all the variables tested. Figure 4 shows the result of this statistical analysis.

As can be seen, PCA groups the variables under study into different clusters depending on the percentage of ethanol of the extraction solvent in clockwise direction. In the first group, only gallic acid was included (GA), as it was the only compound extracted significantly when water was used as extraction solvent. At the same time as we move along the plane in clockwise direction, we found the following cluster formed by ABTS, DPPH, FRAP, quercetin (Q), TPC, and malvidin-3-glucoside (M3G). Consequently, the antioxidant capacity of the extracts obtained was found to be closely correlated with the content of quercetin and malvidin-3-glucoside. The next cluster in the PCA was formed by catechin (CAT), quercetin derivative (QG) and the unknown anthocyanin (ANT). These compounds also show an important correlation with the variables found in the previous group, although they are more closely related between themselves and for that reason, they constitute an independent group. Queiroz et al. [38] isolated five phenolic compounds from grape stem extracts and analyzed their antioxidant capacity. These authors found that malvidin-3-*O*-glucoside and malvidin-3-*O*-(6-*O*-caffeoyl)-glucoside along with quercetin-3-*O*-glucoside were the compounds with greatest antiradical activity. If the relationship between structure and antioxidant capacity is considered, it is well known that the presence of a catechol group in the B ring of flavonoids is the most determinant structural factor on antioxidant activity as it confers stability to the radical formed after the reaction with the free radical [49]. In addition, the presence of a double bond in position 2-3 in conjunction with the 4-oxo group in the carbonyl of ring C and the presence of hydroxyl groups in positions three and five are structural aspects that provide greater antioxidant potential [50]. Consequently, quercetin is one of the compounds with greater antioxidant potential, higher than its glycosylated derivatives, and anthocyanins can be equipotent to quercetin [49], which explains the results obtained in our PCA analysis.

Resveratrol (RSV) and the total content of flavonoids constitute the last cluster. This group is characterized by requiring a high content of ethanol in the extraction solvent, and consequently it is inversely correlated with gallic acid cluster. For that reason, these clusters are in opposite positions in the PCA plane. Likewise, the absence of correlation between resveratrol and antioxidant capacity is confirmed, independently of the spectrophotometric method used, since these variables are found orthogonally arranged in the PCA plane.

Finally, in Figure 4 it is seen that the correlation between the total flavonoids content and all the flavonoids in general is not very high. This could indicate that the spectrophotometric method selected for the total flavonoids determination is not very specific or shows some interferences in these type of samples, so it is not very recommendable for these kind of assays. In this sense, the correlation existing between resveratrol and the total flavonoids variable is also surprising since, *a priori*, it would not seem to make much sense and so, again points out that the AlCl_3_ method for the determination of total flavonoids is not suitable for analyzing complex matrices such as grape stem extracts.

## 4. Conclusions

In Mazuelo stem it was observed that when water was used as extraction solvent gallic acid was the main component of the resulting extract, while when ethanol 50% was used instead, catechin and a quercetin derivative were the main extracted compounds. The PCA analysis showed that both quercetin and malvidin-3-glucoside were closely correlated with the antioxidant capacity of the extract, while stilbenes barely correlate. In general, ethanol concentration was the most determinant parameter on the final composition of the extracts, though several interferences between temperature, ratio solid/solvent and ethanol content were found. The extractions conditions that allow maximum polyphenolic content and antioxidant activity of grape stem extracts were 50% ethanol and 40 °C. However, the selection of the most adequate extraction conditions will depend on the desired compound to be extracted. In all cases food quality ethanol (from 0% to 100% in water) was the selected extraction medium, as its use allows the exploitation of the grape stem extracts in food and pharma industries as healthy valuable ingredients. Once the influence between extraction conditions (mainly solvent and temperature) and extract composition is well established, further studies should be conducted in order to explore how the use of more intensive techniques such as high temperature, ultrasounds or microwave extraction processes could affect the composition of the extract. In this way, a complete and thoroughly map of extraction methods could be described for different applications.

## Figures and Tables

**Table 1:** Estimations of the hierarchical linear model for the spectrophotometry parameters.

Parameter	Int ^a^	Temp	Ratio	% Ethanol				Interactions
				25%	50%	75%	100%	
**ABTS**	0.1 *	0.02	−0.02	0.1 *	0.17 *	0.12 *	0.01	Ratio −50% ethanol: −0.05 *
**DPPH**	0.05 *	0.02 *	−0.02	0.05 *	0.09 *	0.06 *	0.01	Ratio −50% ethanol: −0.04 *
**FRAP**	0.04 *	0.012	−0.011	0.04 *	0.09 *	0.06 *	0.005	
**TPC**	0.07 *	0.005	−0.012	0.04 *	0.11 *	0.09 *	0.002	Temp. −25% ethanol: 0.03 * Temp. −50% ethanol: 0.03 * Temp. −75% ethanol: 0.03 *
**TF**	0.19 *	0.004	−0.08 *	0.21 *	0.53 *	0.96 *	0.7 *	Temp. −25% ethanol: 0.1 * Temp. −75% ethanol: 0.17 *

^a^ Intercept: 0% ethanol, 25 °C, ratio 1:100; * *p* < 0.05.

**Table 2:** Phenolic compounds detected in grape stems extracts: Chemical structure, retention time (RT), and maximum absorption wavelength (λ_max_).

Phenolic Compound	Chemical Structure	RT (min)	λ_max_ (nm)
Gallic acid	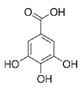	5.9 ± 0.1	272
(+)-Catechin	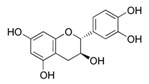	16.3 ± 0.4	279
Quercetin	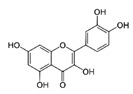	41.6 ± 0.3	369
Quercetin-3-glucuronide *	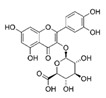	28.8 ± 0.3	355
Quercetin-3-glucoside *	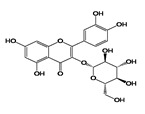	28.2 ± 0.3	354
Malvidin-3-glucoside	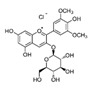	22.5 ± 0.4	526
Anthocyanidin	unknown	35.8 ± 0.4	533
*trans*-Resveratrol	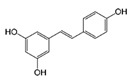	37.8 ± 0.3	306
*trans*-ε-Viniferin	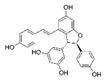	44.1 ± 0.2	324

* Both quercetin-3-glucoside and quercetin-3-glucuronide show similar spectra and retention times, so it was not possible to differentiate them in samples.

**Table 3:** Phenolic composition (μg/g dry matter) of the different extracts obtained from Mazuelo grape stems.

Phenolic Compounds	% Ethanol	Ratio 1:100		Ratio 1:50	
		25 °C	40 °C	25 °C	40 °C
***Phenolic acids***					
**Gallic acid**	0% 25% 50% 75% 100%	197 ± 36 97 ± 5 65 ± 1 63 ± 3 46 ± 5	281 ± 29 83 ± 4 106 ± 47 80 ± 7 61 ± 9	187 ± 15 102 ± 3 59 ± 3 50 ± 10 43 ± 6	310 ±85 81 ± 2 104 ± 49 75 ± 5 59 ± 4
***Flavonoids***					
**Catechin**	0% 25% 50% 75% 100%	296 ± 68 416 ± 26 436 ± 41 431 ± 52 349 ± 38	357 ± 43 542 ± 34 696 ± 359 490 ± 51 413 ± 52	225 ± 36 375 ± 31 392 ± 35 337 ± 81 322 ± 40	332 ± 96 490 ± 65 710 ± 377 473 ± 63 454 ± 36
**Quercetin**	0% 25% 50% 75% 100%	nd 38 ± 2 19 ± 2 16 ± 2 8 ± 2	nd 29 ± 5 23 ± 11 17.1 ± 0.6 11.4 ± 0.9	nd 37 ± 2 18 ± 1 12 ± 2 8 ± 1	nd 30 ± 3 24 ± 12 17 ± 1 12.0 ± 0.3
Quercetin derivative *	0% 25% 50% 75% 100%	149 ± 26 258 ± 20 365 ± 31 372 ± 26 160 ± 14	146 ± 9 325 ± 2 471 ± 213 373 ± 34 201 ± 43	96 ± 9 224 ± 12 333 ± 23 301 ± 61 153 ± 32	108 ± 28 297 ± 34 485 ± 243 368 ± 41 221 ± 15
**Malvidin-3-glucoside**	0% 25% 50% 75% 100%	17 ± 2 42 ± 7 54 ± 1 51 ± 1 21 ± 4	18 ± 7 47 ± 3 64 ± 24 47 ± 3 27 ± 1	10 ± 2 32 ± 2 46 ± 1 38 ± 9 19 ± 2	13 ± 5 36 ± 4 57 ± 20 43 ± 3 26 ± 1
**Anthocyanin**	0% 25% 50% 75% 100%	10 ± 2 62 ± 6 94 ± 6 89 ± 8 41 ± 6	11 ± 2 72 ± 5 118 ± 49 83± 5 49 ± 5	5 ± 2 48 ± 4 81 ± 2 69 ± 16 36 ± 2	7 ± 2 53 ± 8 108 ± 45 79 ± 6 50 ± 3
***Stilbenes***					
***trans*-Resveratrol**	0% 25% 50% 75% 100%	nd nd 71 ± 20 125 ± 17 122 ± 16	nd 23 ± 11 150 ± 73 121 ± 9 123 ± 19	nd nd 76 ± 1 97 ± 22 115 ± 6	nd 21 ± 9 162 ± 74 125 ± 16 136 ± 14
***trans*-ε-Viniferin**	0% 25% 50% 75% 100%	nd 119 ± 16 207 ± 26 193 ± 26 157 ± 17	7 ± 1 166 ± 9 301 ± 160 220 ± 19 192 ± 36	nd 91 ± 12 188 ± 10 164 ± 38 155 ± 15	4 ± 1 141 ± 34 310 ± 150 227 ± 29 221 ± 16

nd: Not detected; * Quercetin-derived compound expressed as quercetin-3-glucoside.

**Table 4:** Estimations of the hierarchically linear model applied to phenolic compounds data.

Phenolic Compounds	Int ^a^	Temp	Ratio	% Ethanol				Interactions
				25%	50%	75%	100%	
**GA**	189.1 *	99.2 *	5.9	−88.2 *	−124 *	−125 *	−142 *	Temp. −25% ethanol: 121 * Temp. −50% ethanol: 60 * Temp. −75% ethanol: 83 * Temp. −100% ethanol: 87 *
**CAT**	297 *	58.5	−73	134 *	136.5 *	124 *	47.5	Temp. −50% ethanol: 205 *
**Q**	0.43	−0.9	−0.9	37 *	19 *	15 *	8 *	Temp. −25% ethanol: 8 *
**QG**	151 *	−9	−59	111.2 *	208.5 *	217 *	8.3	Temp. −50% ethanol: 124 *
**M3G**	17 *	1.8	−6.3	27 *	37 *	33 *	4.2	Temp. −50% ethanol: 8.4 *
**ANT**	10.6 *	−0.12	−6	53 *	83 *	76 *	29.6 *	Temp. −50% ethanol: 24 *
**RSV**	3.5	−7	−7	0.33	69 *	114 *	117 *	Temp. −50% ethanol: 82.5 *
**VIN**	6	−5.4	−12.1	117.5 *	199.2 *	180.1 *	148.9 *	Temp. −50% ethanol: 103 *

^a^ Intercept: 0% ethanol, 25 °C, ratio 1:100; * *p* < 0.05.
